# Identification of ribosomal protein family as immune-cell-related biomarkers of NAFLD by bioinformatics and experimental analyses

**DOI:** 10.3389/fendo.2023.1161269

**Published:** 2023-05-19

**Authors:** Gerui Li, Hang Li, Ze Chen

**Affiliations:** ^1^ Department of Geriatrics, Zhongnan Hospital of Wuhan University, Wuhan, China; ^2^ Department of Cardiology, Zhongnan Hospital of Wuhan University, Wuhan, China; ^3^ Institute of Myocardial Injury and Repair, Wuhan University, Wuhan, China

**Keywords:** non-alcoholic fatty liver disease, immune cell, biomarker, bioinformatics, experimental validation

## Abstract

**Background:**

Immune cells play an integral role in the development and progression of non-alcoholic fatty liver disease (NAFLD). This study was to identify immune-cell-related biomarkers for the diagnosis and treatment of NAFLD.

**Methods and findings:**

First, we introduced human liver transcriptome data from the GEO database (GSE48452 and GSE126848) and performed a weighted gene co-expression network analysis (WGCNA) to screen out the modules related to immune cell infiltration and to identify immune-cell-related differentially expressed genes (ICR-DEGs) associated with NAFLD progression. Further, the protein-protein interaction (PPI) network of ICR-DEGs was established to obtain hub genes and subsequently, the expression trend analysis was conducted to identify immune-cell-related biomarkers of NAFLD. Finally, the mRNA expression of biomarkers was validated in a NAFLD mouse model induced by high-fat diet (HFD) feeding. In total, we identified 66 ICR-DEGs and 13 hub genes associated with NAFLD. Among them, 9 hub genes (*CD247, CD74, FCGR2B, IL2RB, INPP5D, MRPL16, RPL35, RPS3A, RPS8*) were correlated with the infiltrating immune cells by the Pearson correlation analysis. Subsequently, 4 immune-cell-related biomarkers (*RPL35*, *RPS3A*, *RPS8*, and *MRPL16*) with the same expression trends in GSE48452 and GSE126848 datasets were identified. These biomarkers were enriched in immune-related pathways and had a good ability to distinguish between NASH and healthy samples. Moreover, we constructed a competing endogenous RNA (ceRNA) network of biomarkers and predicted twenty potential therapeutic drugs targeting RPS3A such as taxifolin and sitagliptin. Finally, experimental validation indicated that the hepatic mRNA expression of *Rpl35*, *Rps3A*, and *Rps8* was significantly decreased in NAFLD mice.

**Conclusions:**

This study identified four ribosomal protein genes (*RPL35*, *RPS3A*, *RPS8*, and *MRPL16*) as immune-cell-related biomarkers of NAFLD, which may actively participate in the immune processes during NAFLD progression and could serve as potential targets for the diagnosis and treatment of NAFLD.

## 1 Introduction

Non-alcoholic fatty liver disease (NAFLD) encompasses a wide spectrum of liver pathologies ranging from simple steatosis (nonalcoholic fatty liver, NAFL), to nonalcoholic steatohepatitis (NASH), which is characterized by hepatocyte ballooning and lobular inflammation (with or without liver fibrosis), to more advanced stages including liver cirrhosis and hepatocellular carcinoma (HCC) (1). In parallel with the epidemics of obesity and type 2 diabetes mellitus (T2DM), NAFLD has emerged as the most common liver and metabolic disease worldwide, affecting approximately 25% of the global population in 2018 (i.e., 1.7 billion individuals) (2, 3). More alarmingly, the burdens caused by NAFLD are projected to rise rapidly over the next decade (4). Although simple steatosis generally is not associated with a substantial risk of liver-related adverse outcomes, up to 20%-30% of patients with NAFL develop NASH, which can lead to cirrhosis and end-stage liver diseases (1, 5). Notably, NASH becomes the fastest-growing cause of HCC in the USA and Europe (6) and is emerging as a leading etiology for liver transplantation (7). Moreover, NAFLD is now regarded as a multisystem metabolic disease and is closely associated with increased risks of extrahepatic complications such as cardiovascular disease (CVD) and chronic kidney disease (8, 9). Albeit an increasing number of early-stage clinical trials, unfortunately, no specific drugs for NAFLD have been approved so far (10, 11). To date, clinical management is restricted to lifestyle interventions, which are difficult to sustain (2). Thus, there is an imperative need to extensively investigate the pathogenesis of NAFLD and to identify specific molecular biomarkers for the diagnosis and treatment of this burdensome disease.

NAFLD is considered a metabolic disorder; however, during the progression of NAFLD, the immune system plays a pivotal role (12, 13). Indeed, the liver is a crucial immunological organ. Inflammation in NAFLD is triggered by intrahepatic factors (e.g., excess metabolites, lipotoxicity, oxidative stress) and extrahepatic factors (e.g., gut-liver axis, adipose tissue inflammation), leading to distinct immune-mediated alterations in NAFLD (13, 14). A wealth of data has indicated that the innate immune mechanism is a key driver of hepatic inflammation and other pathological changes during NAFLD progression, such as insulin resistance, lipid accumulation, and fibrosis (14, 15). These robust innate immune reactions are intrinsic to the liver. Kupffer cells (KCs), dendritic cells (DCs), and lymphocytes residing in the liver form a coordinated innate immune network. Hepatocytes and liver sinusoidal endothelial cells (LSECs) are not formally innate immune cells, but they can exert immune cell function when under stress (12, 15). These cells can sense the intrahepatic and extrahepatic stimuli and translate those signals into immune responses, leading to pathological alterations during the progression of NAFLD (15). Although innate immunity represents an integral element in supporting liver inflammation, accumulating evidence indicates the important role of adaptive immunity mediated by lymphocytes (CD4+ T cells, CD8+ T cells, and B cells) as an additional factor that promotes hepatic inflammation in NASH (16, 17). Given the key role of the immune cells in the progression of the disease, identifying immune-cell-related molecular targets is crucial for the diagnosis and treatment of NAFLD.

Bioinformatics methods have been widely used to mine transcriptome data to elucidate the pathogenic mechanisms of diseases and to screen out key molecular targets for diagnosis and treatment (18). In the present study, transcriptome data of NAFL, NASH, and healthy human liver samples were obtained from the Gene Expression Omnibus (GEO) database (GSE48452 and GSE126848). Using a series of bioinformatics analysis methods such as weighted gene co-expression network analysis (WGCNA) and protein-protein interaction (PPI) network analysis, we systematically mined immune-cell-related biomarkers of NAFLD and analyzed their potential biological functions, ability to distinguish between disease and normal samples, and correlation with infiltrating immune cells and NAFLD phenotypes. We further validated the expression of biomarkers in a NAFLD mouse model induced by a high-fat diet (HFD). Moreover, we constructed a competing endogenous RNA (ceRNA) network and a transcription factor (TF)-mRNA regulatory network of biomarkers and predicted potential therapeutic drugs targeting these molecules. Overall, four immune-cell-related biomarkers (*RPL35*, *RPS8*, *RPS3A*, and *MRPL16*) were finally screened out, all of which belonged to the ribosomal protein genes. These biomarkers may actively participate in the immune process during NAFLD progression and can serve as potential targets for the diagnosis and treatment of NAFLD.

## 2 Materials and methods

### 2.1 Data acquisition

In total, GSE48452 and GSE126848 datasets of human liver tissue samples, including NAFL, NASH, and healthy control (HC) groups, were collected from the Gene Expression Omnibus (GEO, https://www.ncbi.nlm.nih.gov/geo/) database, which consisted of clinical features and gene expression profiles. The GSE48452 dataset (platform: GPL11532) included 14 HC, 14 NAFL, and 18 NASH cases. The GSE126848 dataset (platform: GPL18573) included 14 HC, 15 NAFL, and 16 NASH cases. In addition, GSE107231 dataset (platform: GPL20115) of human liver tissues from 5 NAFLD patients and 5 HCs was downloaded from the GEO database and used as an external validation dataset.

### 2.2 Analysis of differential genes

The ‘limma’ R package (19) was used to obtain the differentially expressed genes (DEGs) between the NASH group and the HC group in GSE48452 dataset. The *P* value < 0.05 was determined as the significance criteria. A volcano plot was applied to show DEGs by ‘ggplot2’ package (20). The top 50 DEGs were visualized by heatmap. Meanwhile, the same method was used to screen out DEGs between the NAFL group and the HC group in GSE48452 dataset.

### 2.3 Weighted gene co-expression network analysis

The ‘immunedeconv’ package (version 2.0.4) was used to calculate the infiltration ratio of seven immune cells (B cell, macrophage, CD4+ T cell, CD8+ T cell, endothelial cell, uncharacterized cell, and NK cell). WGCNA was performed using the ‘WGCNA’ package (version 1.69) to build a gene co-expression network (21). The proportion of differential immune cells was considered a clinical trait. Firstly, we clustered the samples and removed outliers to ensure the accuracy of the analysis. Subsequently, sample dendrogram and trait heatmap were constructed, and the optimal soft threshold was determined. The similarity between genes was calculated according to the adjacency, and the phylogenetic tree between genes was obtained. The modules were segmented *via* dynamic tree cutting algorithm, and the minModuleSize was 100. We focused on the correlation between modules and clinical traits and screened out the module most relevant to differential immune cells as the key module (*P* < 0.05).

### 2.4 Identification of ICR-DEGs and functional network analysis

Immune cell-related differentially expressed genes (ICR-DEGs) were identified with an intersection of NASH-DEGs, NAFL-DEGs, and key module genes using the ‘VennDiagram’ package (22). Gene Ontology (GO) and Kyoto Encyclopedia of Genes and Genomes (KEGG) enrichment analysis of ICR-DEGs was performed using R package ‘clusterProfiler (23). *P* < 0.05 was used as screening criteria. PPI network which depicts the interactions among representative ICR-DEGs was generated using STRING website (https://string-db.org). Then the topological features of network were visualized using Cytoscape (version 3.7.2) (24). Genes with a degree ≥ 5 were identified as hub genes. Finally, the correlation between hub genes and immune cells with significant differences was analyzed by the Pearson method.

### 2.5 Receiver operating characteristic curve and nomogram creation

Principal component analysis (PCA) was conducted between the NASH/NAFL group and the HC group. Moreover, the ROC curve was plotted to evaluate the diagnostic value of hub genes in GSE48452 dataset by pROC (version 1.16.2) package (25). In addition, the nomogram comprising the key genes was drawn using R ‘rms’ package (26). The corresponding calibration curve was plotted to appraise the precision and reliability of the nomogram model prediction. Furthermore, the decision curve analysis (DCA) curve was plotted by ‘rmda’ package.

### 2.6 Gene set enrichment analysis and correlation analysis with clinical features

The KEGG pathways enriched by key genes were identified by gene set enrichment analysis (GSEA) according to the ‘clusterProfiler’ package (version 3.16.1) (27). Age, body mass index (BMI), leptin, adiponectin, leptin to adiponectin ratio (LAR), liver fat, inflammation, fibrosis, and NAFLD activity score (NAS) were included in the Spearman analysis to explore the correlation between key gene expression and clinical and pathological features. We also compared the expression levels of key genes across different NAS subgroups.

### 2.7 Competitive endogenous RNA regulatory network

The competing endogenous RNA (ceRNA) regulatory networks constructed in the present study mainly included lncRNA-miRNA-mRNA relationship pairs. The miRNAs associated with key genes were screened by the Starbase database (https://starbase.sysu.edu.cn/), and the screening criterion was low stringency ≥ 1. The lncRNAs targeting miRNAs were predicted in the starbase database. The lncRNA-miRNA-mRNA networks were constructed by Cytoscape software (version 3.8.2). Moreover, in order to visualize the regulatory relationships of gene transcription, NetworkAnalyst 3.0 (https://www.networkanalyst.ca/) was applied to predict the transcription factors (TFs) of key genes, and the TF-mRNA regulation network was constructed by Cytoscape software (version 3.8.2).

### 2.8 Drug prediction targeting key genes

In order to explore the potential therapeutic drugs for NAFLD related to biomarkers, targeted drugs associated with key genes were predicted by the CLUE (https://clue.io/command) online database.

### 2.9 Animals and treatments

Twenty-four 8-week-old male C57BL/6J mice were housed under standard conditions (room temperature: 23 ± 2°C; 12 h light/dark cycle) with free access to water and standard laboratory food. After one week of acclimation, all mice were randomly divided into a normal chow (NC) group and a high-fat diet (HFD) group (n = 12 per group), each of which was fed a standard laboratory food or a HFD (60 kcal% fat; D12492, Research Diets, New Brunswick, NJ, USA). The NAFLD mouse model was constructed by 12 weeks of HFD feeding (28–30). After 12 weeks of feeding, all mice were anesthetized with 2% isoflurane and euthanized. The serum and liver samples were collected.

### 2.10 Histology, blood chemistry, and hepatic triglyceride levels

For histological analysis, formalin-fixed mouse liver tissues were processed, and paraffin sections of 5-μm thickness were cut and stained with hematoxylin-eosin (H&E). NAS was used to assess the histological characteristics (31). Serum alanine transaminase (ALT) levels were detected using an ADVIA 2400 Chemistry System analyzer (Siemens, Tarrytown, NY, USA) according to the manufacturer’s instructions. TG contents in the liver were measured by a commercial kit (#290-63701; Wako, Tokyo, Japan) according to the manufacturer’s instructions.

### 2.11 Total RNA extraction and real-time quantitative PCR analysis

Total RNA was extracted with Trizol (No. 15596026, Thermo Fisher Scientific, Waltham, MA, USA) and then reverse-transcribed into cDNA by using the HiScript III RT SuperMix for qPCR (+gDNA wiper) (No. R323-01, Vazyme Biotech, Nanjing, Jiangsu, China) following the manufacturer’s instructions. qPCR was conducted with the QuantStudio^®^ 5 Real-Time PCR System (Thermo Fisher Scientific, Waltham, MA, USA) using Cham Q ™ Universal SYBR^®^ qPCR Master Mix (No. Q712-02, Vazyme Biotech, Nanjing, Jiangsu, China). Expression levels of target genes were calculated by the 2^-ΔΔCt^ method and normalized relative to glyceraldehyde-3-phosphate dehydrogenase (*Gapdh*).

### 2.12 Statistical analyses

All of the analyses were performed using R (V. 4.2.2) statistical software. Quantitative data in the experimental validation analyses were presented as mean ± standard error of mean (S.E.M.). D’Agostino & Pearson normality test was used to evaluate if data followed a parametric or non-parametric distribution. For parametric data between the NC and HFD groups, a two-tailed Student’s t-test was applied to analyze differences. For datasets with a skewed distribution, the Mann–Whitney U test was utilized for two group comparisons. A *P* value < 0.05 (two-tailed) was considered statistically significant.

## 3 Results

### 3.1 Identification of DEGs

**Figure 1:**
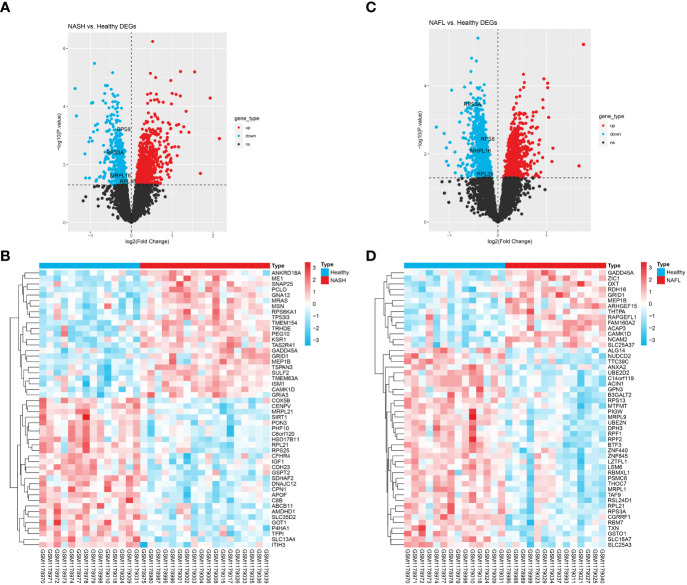
Identification of differentially expressed genes (DEGs). **(A)** DEGs between the NASH group and the healthy control group in GSE48452, with upregulated genes indicated in red and downregulated genes in blue in the volcano plot. **(B)** Heatmap showing the top 50 NASH-DEGs. **(C)** DEGs between the NAFL group and the healthy control group in GSE48452, with upregulated genes indicated in red and downregulated genes in blue in the volcano plot. **(D)** Heatmap showing the top 50 NAFL-DEGs. *P<0.05. NS, non-significant.

As shown in **Figure 1A**, 1766 NASH-DEGs, including 918 up-regulated genes and 848 down-regulated genes, were identified between the NASH group and the HC group. The top 50 NASH-DEGs were visualized by heatmap (**Figure 1B**). Similarly, 2024 NAFL-DEGs (818 upward and 1206 downward) were obtained between the NAFL group and the HC group (**Figure 1C**). The top 50 NAFL-DEGs were visualized by heatmap (**Figure 1D**).

### 3.2 Screening of differential immune cell-related modules

**Figure 2:**
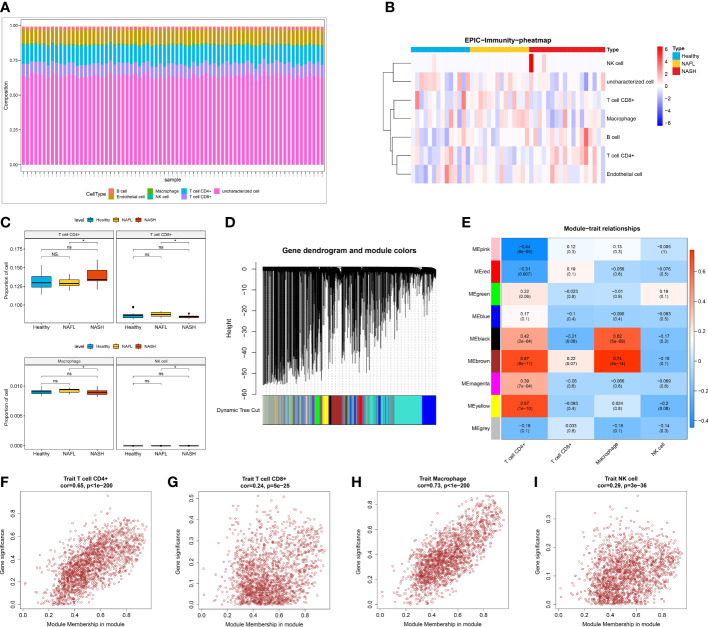
Screening of differential immune cell-related modules. **(A)** The infiltration of seven immune cells (B cell, macrophage, CD4+ T cell, CD8+ T cell, endothelial cell, uncharacterized cell, and NK cell). **(B)** Heatmap showing the infiltration abundance of seven immune cells in healthy control, NAFL, and NASH groups. **(C)** The infiltration ratio of CD4+ T cell, CD8+ T cell, macrophage, and NK cell in healthy control, NAFL, and NASH groups. **(D)** Gene dendrogram and module colors in WGCNA. **(E)** Trait heatmap showing the correlation between genes within modules and immune cells. **(F–I)** The scatter plot of the correlation between genes within the brown module and CD4+ T cell **(F)**, CD8+ T cell **(G)**, macrophage **(H)**, and NK cell **(I)**.

To investigate the features of the immune cells in NAFLD, a comprehensive heatmap was generated to illustrate their expression patterns. Among 7 immune cells, CD4+ T cell and CD8+ T cell showed higher abundance compared with other immune cells (**Figure 2A**). We also observed that the infiltration abundance of 4 immune cells (CD4+ T cell, CD8+ T cell, macrophage, and NK cell) was significantly different between the NASH and NAFL groups (**Figures 2B, C** **)**. To seek out key module genes associated with four differentially infiltrating immune cells (CD4+ T cell, CD8+ T cell, macrophage, and NK cell), we conducted a WGCNA. The results of sample clustering indicated that there were no outlier samples. The sample dendrogram and trait heatmap were shown in **Supplementary Figure 1**. The optimal soft threshold was chosen 10 to ensure the network was close to scale-free distribution (**Supplementary Figure 2**). A total of 9 modules were obtained by the dynamic tree cut algorithm (**Figure 2D**). Among the 9 modules, the brown module was most closely related to 4 differentially infiltrating immune cells (**Figure 2E**). The correlation coefficient between the genes within the brown module and CD 4+ cell was 0.65, CD 8+ T cell was 0.24, macrophage was 0.73, and NK cell was 0.29 (*P* < 0.05, **Figures 2F–I**). Therefore, a total of 1862 differential immune cell-related genes within the brown module were obtained.

### 3.3 Identification of ICR-DEGs and biomarkers

**Figure 3:**
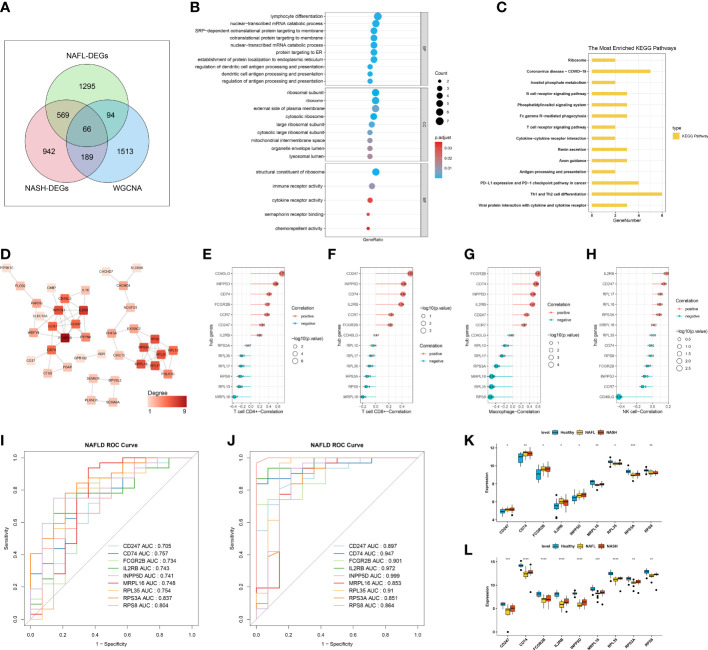
Identification of immune-cell-related differentially expressed genes (ICR-DEGs) and biomarkers. **(A)** Venn diagram obtained 66 ICR-DEGs by overlapping NASH-DEGs, NAFL-DEGs, and key module genes correlated with 4 immune cells. **(B)** The top GO items under biological process (BP), molecular function (MF), and cellular component (CC). **(C)** The most enriched KEGG pathways. **(D)** Protein-protein interaction (PPI) network of ICR-DEGs. **(E–H)** Pearson correlation analysis between the hub genes and CD4+ T cell **(E)**, CD8+ T cell **(F)**, macrophage **(G)**, and NK cell **(H)**. **(I)** Receiver operator characteristic (ROC) curve of 9 hub genes in dataset GSE48452. **(J)** ROC curve of 9 hub genes in the external validation dataset GSE126848. **(K, L)** The expression level of 9 hub genes in healthy control, NAFL, and NASH groups of GSE48452 **(K)** and GSE126848 **(L)**, respectively.

In total, 66 ICR-DEGs were obtained by overlapping NASH-DEGs, NAFL-DEGs, and key module genes correlated with 4 differentially infiltrating immune cells **(** **Figure 3A** **)**. In order to uncover potential mechanisms for ICR-DEGs associated with NAFLD, we proceeded with functional enrichment analysis. The top GO items under each classification were shown in **Figure 3B**. We observed that the above ICR-DEGs were principally linked to ‘lymphocyte differentiation’, ‘nuclear−transcribed mRNA catabolic process’, and ‘cotranslational protein targeting to membrane’. In addition, the KEGG results indicated that these ICR-DEGs were mainly enriched in the ‘ribosome’, ‘B cell receptor signaling pathway’, and ‘T cell receptor signaling pathway’ (**Figure 3C**). Moreover, the PPI network of ICR-DEGs was constructed, including 38 nodes and 64 edges (**Figure 3D**). Thirteen hub genes were obtained by analyzing the topological structure of genes (**Supplementary Table 1**). The Pearson correlation analysis was performed between the hub genes and key immune cells, and 9 hub genes (*CD247*, *CD74*, *FCGR2B*, *IL2RB*, *INPP5D*, *MRPL16*, *RPL35*, *RPS3A*, and *RPS8*) exhibited significant correlations with CD4+ T cell, CD8+ T cell, and macrophage (**Figures 3E–H**). As shown in **Supplementary Figure 3**, PCA suggested that the 9 hub genes could distinguish control and disease samples, indicating the hub genes had a certain diagnostic ability for NAFL and NASH. ROC curve results showed that all 9 hub genes had good diagnostic values (AUC > 0.7) for NAFLD (**Figure 3I**, **Supplementary Table 2**). Next, we observed that the ROC curve results of the external validation dataset (GSE126848) were consistent with those of the GSE48452 dataset (**Figure 3J**, **Supplementary Table 2**). At the level of gene expression, we found that *MRPL16*, *RPL35*, *RPS3A*, and *RPS8* showed the same expression trend in the two datasets (**Figures 3K, L** **)**. Therefore, we defined these four genes as immune-cell-related key genes associated with NAFLD progression, namely biomarkers.

### 3.4 Diagnostic nomogram model of biomarkers

**Figure 4:**
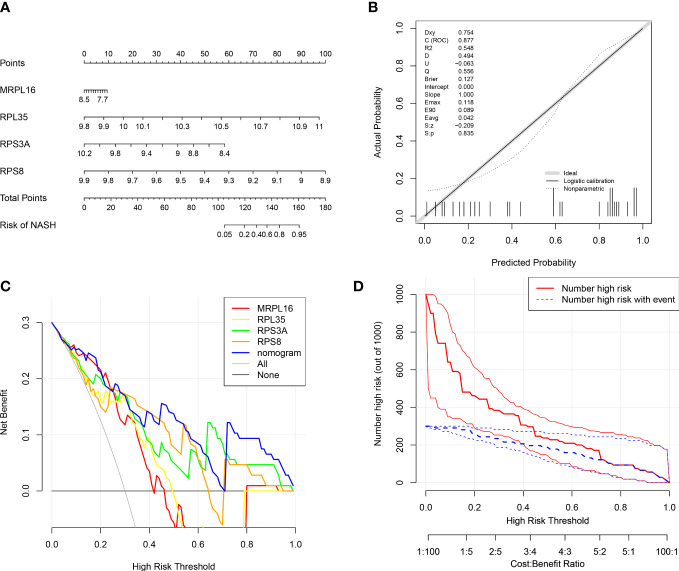
Diagnostic nomogram model of biomarkers. **(A)** Nomogram containing four biomarkers for the diagnosis of NASH. **(B)** Calibration curve of the nomogram model. **(C)** Decision curve analysis (DCA) curve of the nomogram model. **(D)** Clinical influence curve of the nomogram model.

In order to evaluate the role of four biomarkers in NASH diagnosis, the nomogram containing four biomarkers (*MRPL16*, *RPL35*, *RPS3A*, and *RPS8*) was generated (**Figure 4A**), and the calibration curves proved that the performance of the prediction model was effective (**Figure 4B**). In addition, DCA results showed that the nomogram model was clinically feasible (**Figure 4C**). Furthermore, the clinical influence curve further revealed that the nomogram model had accurate prediction ability (**Figure 4D**).

### 3.5 Functional enrichment and correlation analysis between biomarkers and clinical features

**Figure 5:**
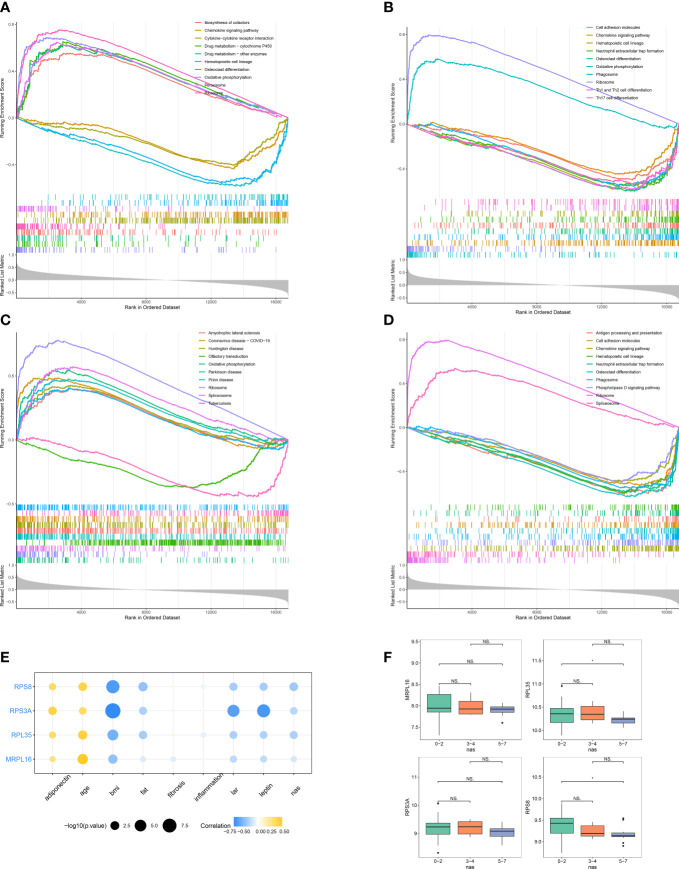
Functional enrichment and clinical correlation analysis based on the biomarkers. **(A–D)** Gene set enrichment analysis (GSEA) of *MRPL16* **(A)**, *RPL35* **(B)**, *RPS3A* **(C)**, and *RPS8* **(D)** in GSE48452. **(E)** Correlation cycle diagram illustrating the relationships of four biomarkers (*MRPL16*, *RPL35*, *RPS3A*, and *RPS8*) with different clinical and pathological features, including adiponectin, age, BMI, liver fat, liver fibrosis, liver inflammation, leptin to adiponectin ratio (LAR), leptin, and NAFLD activity score (NAS) in GSE48452. **(F)** The expression of biomarker genes across the NAS 0-2, 3-4, and 5-7 groups.

All biomarkers (*RPL35*, *RPS8*, *RPS3A*, and *MRPL16*) belonged to the ribosomal protein genes. To further study the potential roles of four biomarkers in NAFLD, we performed GSEA on each biomarker in GSE48452. The results of GSEA showed that ‘ribosome’ pathway was enriched in the groups with a high expression of *MRPL16*, *RPL35*, *RPS3A*, and *RPS8*, while ‘chemokine signaling pathway’ was associated with a low expression of *MRPL16*, *RPL35*, and *RPS8* (**Figures 5A–D**). We also generated a correlation cycle diagram to illustrate the relationships of four biomarkers (*MRPL16*, *RPL35*, *RPS3A*, and *RPS8*) with different clinical and pathological features. The four biomarkers were negatively correlated with five factors (BMI, leptin, LAR, NAS, and liver fat), whereas positively correlated with age and adiponectin (**Figure 5E**). In addition, the expression of *RPL35* and *RPS8* was significantly lower in the NAS 5-7 group compared to the NAS 0-2 group (*P* < 0.05, **Figure 5F**).

### 3.6 The ceRNA network and TF-mRNA regulatory network based on four biomarkers

**Figure 6:**
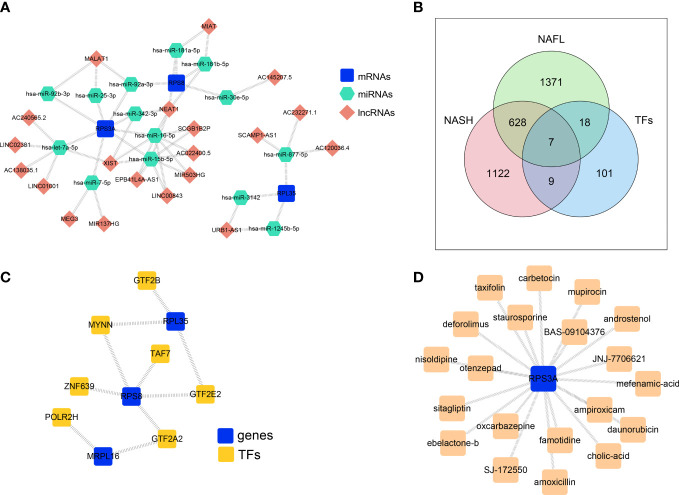
The ceRNA network and TF-mRNA regulatory network based on four biomarkers. **(A)** ceRNA network based on *MRPL16*, *RPL35*, *RPS3A*, and *RPS8*. **(B)** Venn diagram overlapping the predicted transcription factors of 4 biomarkers, NASH-DEGs, and NAFL-DEGs. **(C)** The TF-mRNA regulatory network based on biomarkers. **(D)** Key gene-drug network showing the compounds targeting the protein encoded by *RPS3A.*

To explore the regulatory mechanisms of biomarkers, the ceRNA network based on *MRPL16*, *RPL35*, *RPS3A*, and *RPS8* was constructed. The two pair groups (lncRNA-miRNA pairs and mRNA-miRNA pairs) were matched to yield a ceRNA network ‘lncRNA-miRNA-mRNA’, which consisted of 20 lncRNA, 3 mRNA, and 14 miRNA. This network had 37 nodes and 51 edges (**Figure 6A**). The ceRNA network showed that AC120036.4 might regulate *RPL35* through hsa-miR-877-5p, AC240565.2 or LINC02381 might regulate *RPS3A* through hsa-let-7a-5p, and MIAT might regulate *RPS8* through hsa-miR-181a-5p or hsa-miR-181b-5p. Furthermore, the transcription factors of 4 biomarkers were predicted, and seven transcription factors (GTF2B, MYNN, TAF7, ZNF639, POLR2H, GTF2E2, GTF2A2) that were significantly and differentially expressed in the NASH and NAFL groups were obtained (**Figure 6B**). The TF-mRNA regulatory network had 10 nodes and 10 edges (**Figure 6C**), in which *RPL35* and *RPS8* were mutually associated with MYNN.

### 3.7 The key gene-drug network

In order to explore the potential therapeutic drugs for NAFLD related to biomarkers, the compounds targeting the protein encoded by *RPS3A* were identified (**Figure 6D**, **Supplementary Table 3**). There were 20 drugs with potential therapeutic effects on RPS3A, including taxifolin, sitagliptin, otenzepad, famotidine, and JNJ-7706621, etc.

### 3.8 Validation of the immune-cell-related biomarkers

**Figure 7:**
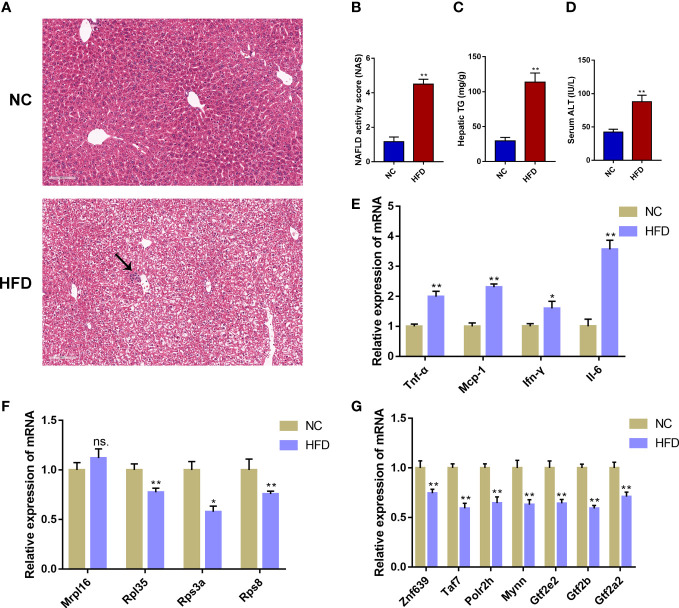
Validation of the immune-cell-related biomarkers in a NAFLD mouse model. **(A)** H&E staining of mouse liver tissue from the normal chow (NC) group and the high-fat diet (HFD) group. The black arrow indicates infiltrated immune cells. **(B)** NAFLD activity score (NAS). **(C)** Hepatic triglyceride (TG) concentrations. **(D)** Serum alanine aminotransferase (ALT) levels. **(E)** Relative mRNA expression level of inflammatory marker genes. **(F)** Relative mRNA expression level of immune-cell-related biomarker genes. **(G)** Relative mRNA expression level of the predicted transcription factors. Mean ± S.E.M., *n* = 12. ^*^ *P<*0.05, ^**^ *P<*0.01 *vs.* the NC group.

To further verify the reliability of the obtained immune-cell-related biomarkers in NAFLD, we assessed the expression of these biomarkers in an external validation dataset (GSE107231) and a NAFLD mouse model induced by 12-week HFD feeding. In GSE107231, the expression of *RPL35*, *RPS3A*, and *RPS8* was significantly decreased in NAFLD livers compared to the healthy controls **(** **Supplementary Figure 4** **)**. In animal experiments, the H&E staining showed marked hepatic steatosis and sporadic inflammation in the HFD group **(** **Figure 7A** **)**. Compared with the NC group, the HFD group showed significantly increased NAS, hepatic TG levels, and serum ALT concentrations (*P* < 0.01, **Figures 7B–D**, **Supplementary Table 4**). Moreover, the mRNA expression of inflammatory markers (*Tnf-α*, *Mcp-1*, *Ifn-γ*, and *Il-6*) was significantly higher in the HFD group compared to that in the NC group (*P* < 0.05, *P* < 0.01, **Figure 7E**). The validation of the mRNA expression of 4 biomarkers showed that the expression of *Rpl35*, *Rps3a*, and *Rps8* was significantly decreased in the HFD group compared to the NC group (*P* < 0.05, *P* < 0.01), which was consistent with the human transcriptome data. However, the expression of *Mrpl16* was not significantly changed **(** **Figure 7F** **)**. Further detection on the mRNA expression of the 7 predicted TFs (*Znf639*, *Taf7*, *Polr2h*, *Mynn*, *Gtf2e2*, *Gtf2b*, *Gtf2a2*) showed that the expression of all 7 TFs was significantly decreased in the HFD group compared to the NC group **(** **Figure 7G** **)**.

## 4 Discussion

NAFLD has become the most common liver and metabolic disease worldwide and placed a tremendous burden on public health (2). Immune cells and immune-related genes play an integral role during the progression of NAFLD (13). Identifying immune-cell-related molecular biomarkers is crucial for the diagnosis and treatment of NAFLD. In the present study, we applied a series of bioinformatics methods to analyze the transcriptome data and found that the infiltration abundance of 4 immune cells (CD4+ T cell, CD8+ T cell, macrophage, and NK cells) was significantly different between the NASH and NAFL samples. Moreover, four immune-cell-related biomarkers (*RPL35*, *RPS8*, *RPS3A*, and *MRPL16*) were screened out, all of which belong to the ribosomal protein genes. Validation in both an external GEO dataset and in a mouse model showed that the hepatic mRNA expression of *RPL35*, *RPS3A*, and *RPS8* was significantly decreased in NAFLD. These immune-cell-related biomarkers might actively participate in the immune process during NAFLD progression and could serve as potential targets for the diagnosis and treatment of NAFLD.

Immune cell infiltration exerts critical effects during the development and progression of NAFLD (15). Cell type identification is very helpful for deciphering the pathogenesis of various diseases (32). In the present study, we systematically estimated the relative infiltration abundances of different immune cell types by transcriptome analysis and found differences in infiltration abundances of 4 immune cells (CD4+ T cell, CD8+ T cell, macrophage, and NK cells) between the NASH and NAFL liver samples. These immune cells are considered important effector cells in NAFLD, and their cell frequencies and roles in the pathogenesis of NAFLD have been summarized in some elegant reviews (12, 13, 15, 33). In this study, CD4+ T cells showed the highest infiltration abundance and their infiltration was significantly higher in the NASH group compared to the NAFL group. The balance of CD4+ T helper (Th) cells, which can be broadly categorized into Th1, Th2, Th17, and regulatory T cells (Tregs), is important to maintain hepatic immune tolerance (13). The imbalance of regulatory and effector T helper cells is a hallmark of multiple chronic liver diseases (34). Many studies have indicated that Th1 and Th17 cells are increased in NASH patients (35). An animal study found that human CD4+ T cells accumulated in the liver of a humanized mouse model fed a high-fat high-calorie diet, and depletion of these cells alleviated hepatic inflammation and fibrosis (36). Moreover, IFN-γ-expressing CD4+ T cells were enriched in NASH, and methionine/choline deficient (MCD) diet feeding induced milder steatohepatitis and decreased inflammatory macrophage infiltration in mice deficient in IFN-γ compared to the control mice (37, 38). It should be noted that whether an altered subset of immune cells is a cause or a consequence of NAFLD progression is difficult to figure out. The progression of NAFLD driven by immune cells is a multistage process, involving the interactions of different immune cells (13). Thus, further organ- and cell-specific loss-of-function experiments, combined with appropriate animal models, are urgently needed to decipher the complicated roles of different immune cell types during the progression of NAFLD.

Four genes (*RPL35*, *RPS8*, *RPS3A*, and *MRPL16*) were screened out as immune-cell-related biomarkers of NAFLD, among which three genes (*RPL35*, *RPS8*, and *RPS3A*) showed consistent expression trends in a rodent model and three independent human datasets. Intriguingly, all biomarkers belong to the ribosomal protein genes. Ribosomal proteins are typically small and basic proteins containing 50-150 amino acid residues, which play a seminal role in the structure and function of ribosomes or the initiation, elongation, or termination phases of protein translation (39, 40). Although human ribosomes have long been regarded as uniform factories with little regulatory functions, increasing evidence highlighted the expression heterogeneity of ribosomal proteins in association with specific cellular functions (40, 41). Indeed, ribosomal protein genes are differentially expressed across various normal tissues and cell types and are actively involved in specific cellular functions such as cellular metabolism, cell cycle, and signal transduction and development (39, 40). A recent study found that ribosomal protein deficiency induced substantial alterations in gene expression, with the effect of each ribosomal protein varying to different extents (41).

In our results, RPS3A and RPS8 are components of the 40S subunit in the prokaryotic ribosome, RPL35 is a component of the 60S subunit in the prokaryotic ribosome, and MRPL16 is a component of the 39S subunit in the mitochondrial ribosome. RPS3A belongs to the S3AE family of ribosomal proteins. In addition to its ribosomal function, RPS3A can also perform multiple biological functions unrelated to the ribosomes such as cell proliferation, inflammation, and cellular metabolism (42, 43). Several studies have reported that RPS3A is highly expressed in some transformed cells and tumors, and plays a critical role in the regulation of cell proliferation and transformation by exerting extra-ribosomal functions (44–46). High expression of RPS3A correlated with low tumor immune cell infiltration and an unfavorable prognosis in patients with HCC (47). RPS3A over-expressed in HBV-associated HCC could enhance hepatitis B virus X protein (HBx)-induced NF-κB signaling *via* a novel chaperoning activity for aggregation-prone HBx and thereby contribute to hepatitis B-induced oncogenesis (48). Moreover, RPS3A positively regulated the mitochondrial function of human periaortic adipose tissues and was associated with the risk of coronary artery diseases (49). RPS8 belongs to the S8E family of ribosomal proteins. RPS8 deficiency could stimulate cellular apoptosis and significantly inhibit cell proliferation (41). Previous studies have reported that the mRNA expression level of *RPS8* was elevated in pancreatic cancer tissues and associated with poor prognosis (50, 51). In addition, RPS8 was highly expressed in alcohol-associated HCC and associated with tumor progression, thus serving as a potential biomarker and therapeutic target for alcohol-associated HCC (52). RPL35 belongs to the L29P family of ribosomal proteins and interacts with eukaryotic translation elongation factor 2 thereby regulating protein synthesis (53). RPL35 is also reported as a key factor for promoting E2F1 protein synthesis, N-Myc protein stability, and N-Myc-driven oncogenesis (54). Moreover, RPL35 can exert extra ribosomal functions. RPL35 promoted neuroblastoma progression *via* enhanced aerobic glycolysis (55). As for MRPL16, few studies have reported its functions to date and only one study reported that low levels of MRPL16 significantly indicated poor prognosis in breast cancer patients (56). Overall, the above literature suggested that these ribosomal proteins could exert multiple biological functions such as the regulation of immune responses. Thus, the four ribosomal proteins might play an important role in the pathogenesis of various diseases and could serve as potential biomarkers of diseases other than NAFLD such as cancers. However, it should be noted that the functional role of these molecules is still not fully understood. Further gain/loss-of-function experiments are required to investigate the cell-specific functions of these immune-cell-related biomarkers in the pathogenesis of NAFLD and other diseases.

Functional enrichment results showed that ‘ribosome’ pathway was enriched in the groups with a high expression of *MRPL16*, *RPL35*, *RPS3A*, and *RPS8*, while a number of immune-related pathways were associated with a low expression of biomarker genes. For instance, ‘chemokine signaling pathway’, ‘neutrophil extracellular trap formation’, and ‘cell adhesion molecules’ were associated with a low expression of *RPS8* and *RPL35. ‘*Antigen processing and presentation’ was associated with a low expression of *RPS8.* ‘Th1 and Th2 cell differentiation’ and ‘Th17 cell differentiation’ were associated with low-expression of *RPL35*. Moreover, our results demonstrated that the expression of *RPL35* and *RPS8* was significantly lower in the NAS 5-7 group compared to the NAS 0-2 group. These results further supported that these biomarkers may play important roles in immune responses during the progression of NAFLD.

Our study constructed a ceRNA network, which consisted of 20 lncRNA, 3 mRNA, and 14 miRNA. Some of these molecules have been reported to be associated with immune reactions. For example, hsa-let-7a-5p could positively regulate the important innate immune-related genes such as TLR3, RIG-I, and MDA5, thus promoting innate immune responses (57). LncRNA MIAT correlated with immune infiltrates in HCC and targeted miR-411-5p/STAT3/PD-L1 axis mediating HCC immune response (58, 59). miR-181a-5p over-expression could alleviate Treg/Th17 immune imbalance and block allergic rhinitis from developing into asthma (60), while decreased miR-181a-5p expression was associated with impaired NK cell development and function with aging (61). However, the existence and functional role of the predicted ceRNA network in the development and progression of NAFLD requires further validation.

Our results also identified 20 potential drugs that may target RPS3A, among which taxifolin and sitagliptin have been reported to be beneficial against NAFLD. Taxifolin, also known as dihydroquercetin, is a natural bioactive flavonoid that broadly existed in various foods and health supplement products (62). Recent studies found that taxifolin had antioxidant and anti-inflammatory properties and could ameliorate steatohepatitis induced by HFD feeding plus acute ethanol binge by inhibiting inflammatory caspase-1-dependent pyroptosis (62). In addition, taxifolin displays pleiotropic effects for the treatment of the NASH continuum. It could alleviate obesity-induced hepatic steatosis, fibrosis, and tumorigenesis in rodent models (63). Sitagliptin is a selective inhibitor of dipeptidyl peptidase-4 (DPP-4I), which is widely used as a hypoglycemic agent. Animal studies showed that sitagliptin could improve NAFLD in HFD-fed rodents (64, 65). A randomized controlled trial indicated that sitagliptin, combined with metformin, reduced body weight, intrahepatic lipid, and visceral adipose tissue in addition to improving glycemic control in patients with NAFLD and T2DM (66). Nevertheless, whether the effects of taxifolin and sitagliptin on NAFLD were related to *RPS3A* needs further investigation.

In conclusion, this study identified four ribosomal protein genes (*RPL35*, *RPS8*, *RPS3A*, and *MRPL16*) as immune-cell-related biomarkers associated with NAFLD progression, which may help to better understand the role of immune-related genes and cells in the pathogenesis of NAFLD and to develop novel diagnostic and therapeutic strategies. However, this study did not validate the molecular function of these biomarkers and the existence and functional role of the ceRNA network, TF-mRNA network, and key gene-drug network in NAFLD, which is a major limitation. Further *in vivo* and *in vitro* experiments as well as clinical studies are needed to elucidate the functions, the underlying mechanisms, and the translational potential of these biomarkers.

## Data availability statement

The datasets presented in this study can be found in online repositories. The names of the repository/repositories and accession number(s) can be found in the article/**Supplementary Material**.

## Ethics statement

The animal study was reviewed and approved by Experimental Animal Welfare Ethics Committee of Zhongnan Hospital of Wuhan University.

## Author contributions

ZC conceived and designed the study and experiments. GL and HL sorted out the data, performed the data analysis, conducted the validation experiments, and wrote the manuscript. ZC supervised this study and revised the manuscript. All authors contributed to the article and approved the submitted version.

## References

[1] Diehl AM Day C. Cause, pathogenesis, and treatment of nonalcoholic steatohepatitis. N Engl J Med (2017) 377(21):2063–72. doi: 10.1056/NEJMra1503519 29166236

[2] Chalasani N Younossi Z Lavine JE Charlton M Cusi K Rinella M. The diagnosis and management of nonalcoholic fatty liver disease: practice guidance from the American association for the study of liver diseases. Hepatology (2018) 67(1):328–57. doi: 10.1002/hep.29367 28714183

[3] Chen Z Liu J Zhou F Li H Zhang XJ She ZG. Nonalcoholic fatty liver disease: an emerging driver of cardiac arrhythmia. Circ Res (2021) 128(11):1747–65. doi: 10.1161/CIRCRESAHA.121.319059 34043417

[4] Estes C Anstee QM Arias-Loste MT Bantel H Bellentani S Caballeria J. Modeling nafld disease burden in China, France, Germany, Italy, Japan, Spain, united kingdom, and united states for the period 2016-2030. J Hepatol (2018) 69(4):896–904. doi: 10.1016/j.jhep.2018.05.036 29886156

[5] Lindenmeyer CC McCullough AJ. The natural history of nonalcoholic fatty liver disease-an evolving view. Clin Liver Dis (2018) 22(1):11–21. doi: 10.1016/j.cld.2017.08.003 29128051 PMC6130315

[6] Huang DQ El-Serag HB Loomba R. Global epidemiology of nafld-related hcc: trends, predictions, risk factors and prevention. Nat Rev Gastroenterol Hepatol (2021) 18(4):223–38. doi: 10.1038/s41575-020-00381-6 PMC8016738 33349658

[7] Wong RJ Singal AK. Trends in liver disease etiology among adults awaiting liver transplantation in the united states, 2014-2019. JAMA Netw Open (2020) 3(2):e1920294. doi: 10.1001/jamanetworkopen.2019.20294 32022875 PMC12124732

[8] Cai J Zhang XJ Ji YX Zhang P She ZG Li H. Nonalcoholic fatty liver disease pandemic fuels the upsurge in cardiovascular diseases. Circ Res (2020) 126(5):679–704. doi: 10.1161/CIRCRESAHA.119.316337 32105577

[9] Li G Peng Y Chen Z Li H Liu D Ye X. Bidirectional association between hypertension and nafld: a systematic review and meta-analysis of observational studies. Int J Endocrinol (2022) 2022:8463640. doi: 10.1155/2022/8463640 35371259 PMC8970889

[10] Chen Z Yu Y Cai J Li H. Emerging molecular targets for treatment of nonalcoholic fatty liver disease. Trends Endocrinol Metab (2019) 30(12):903–14. doi: 10.1016/j.tem.2019.08.006 31597607

[11] Ferguson D Finck BN. Emerging therapeutic approaches for the treatment of nafld and type 2 diabetes mellitus. Nat Rev Endocrinol (2021) 17(8):484–95. doi: 10.1038/s41574-021-00507-z PMC8570106 34131333

[12] Huby T Gautier EL. Immune cell-mediated features of non-alcoholic steatohepatitis. Nat Rev Immunol (2022) 22(7):429–43. doi: 10.1038/s41577-021-00639-3 PMC8570243 34741169

[13] Peiseler M Schwabe R Hampe J Kubes P Heikenwalder M Tacke F. Immune mechanisms linking metabolic injury to inflammation and fibrosis in fatty liver disease - novel insights into cellular communication circuits. J Hepatol (2022) 77(4):1136–60. doi: 10.1016/j.jhep.2022.06.012 35750137

[14] Chen Z Tian R She Z Cai J Li H. Role of oxidative stress in the pathogenesis of nonalcoholic fatty liver disease. Free Radic Biol Med (2020) 152:116–41. doi: 10.1016/j.freeradbiomed.2020.02.025 32156524

[15] Cai J Zhang XJ Li H. The role of innate immune cells in nonalcoholic steatohepatitis. Hepatology (2019) 70(3):1026–37. doi: 10.1002/hep.30506 30653691

[16] Sutti S Albano E. Adaptive immunity: an emerging player in the progression of nafld. Nat Rev Gastroenterol Hepatol (2020) 17(2):81–92. doi: 10.1038/s41575-019-0210-2 31605031 PMC7222953

[17] Mao T Yang R Luo Y He K. Crucial role of T cells in nafld-related disease: a review and prospect. Front Endocrinol (Lausanne) (2022) 13:1051076. doi: 10.3389/fendo.2022.1051076 36457551 PMC9705593

[18] Sepulveda JL. Using R. And bioconductor in clinical genomics and transcriptomics. J Mol Diagn (2020) 22(1):3–20. doi: 10.1016/j.jmoldx.2019.08.006 31605800

[19] Colaprico A Silva TC Olsen C Garofano L Cava C Garolini D. Tcgabiolinks: an R/Bioconductor package for integrative analysis of tcga data. Nucleic Acids Res (2016) 44(8):e71. doi: 10.1093/nar/gkv1507 26704973 PMC4856967

[20] Gómez-Rubio V. Ggplot2 - elegant graphics for data analysis (2nd edition). J Stat Software Book Rev (2017) 77(2):1–3. doi: 10.18637/jss.v077.b02

[21] Langfelder P Horvath S. Wgcna: an r package for weighted correlation network analysis. BMC Bioinf (2008) 9:559. doi: 10.1186/1471-2105-9-559 PMC2631488 19114008

[22] Li HG Zhao LH Bao XB Sun PC Zhai BP. Meta-analysis of the differentially expressed colorectal cancer-related microrna expression profiles. Eur Rev Med Pharmacol Sci (2014) 18(14):2048–57. 25027346

[23] Yu G Wang LG Han Y He QY. Clusterprofiler: an r package for comparing biological themes among gene clusters. OMICS (2012) 16(5):284–7. doi: 10.1089/omi.2011.0118 PMC3339379 22455463

[24] Chen XM Zhao Y Wu XD Wang MJ Yu H Lu JJ. Novel findings from determination of common expressed plasma exosomal micrornas in patients with psoriatic arthritis, psoriasis vulgaris, rheumatoid arthritis, and gouty arthritis. Discovery Med (2019) 28(151):47–68. 31465725

[25] Robin X Turck N Hainard A Tiberti N Lisacek F Sanchez JC. Proc: an open-source package for r and s+ to analyze and compare roc curves. BMC Bioinf (2011) 12:77. doi: 10.1186/1471-2105-12-77 PMC3068975 21414208

[26] Li G Bing YT Tian ML Yuan CH Xiu DR. Using a nomogram to preoperatively predict distant metastasis of pancreatic neuroendocrine tumor in elderly patients. Chin Med Sci J (2021) 36(3):218–24. doi: 10.24920/003722 34666875

[27] Kumar L EF M. Mfuzz: a software package for soft clustering of microarray data. Bioinformation (2007) 2(1):5–7. doi: 10.6026/97320630002005 18084642 PMC2139991

[28] Qian X Wang T Gong J Wang L Chen X Lin H. Exercise in mice ameliorates high-fat diet-induced nonalcoholic fatty liver disease by lowering Hmgcs2. Aging (Albany NY) (2021) 13(6):8960–74. doi: 10.18632/aging.202717 PMC8034885 33647884

[29] Di Mauro S Salomone F Scamporrino A Filippello A Morisco F Guido M. Coffee restores expression of lncrnas involved in steatosis and fibrosis in a mouse model of nafld. Nutrients (2021) 13(9):2952. doi: 10.3390/nu13092952 34578828 PMC8467439

[30] Casimiro I Stull ND Tersey SA Mirmira RG. Phenotypic sexual dimorphism in response to dietary fat manipulation in C57bl/6j mice. J Diabetes Complications (2021) 35(2):107795. doi: 10.1016/j.jdiacomp.2020.107795 33308894 PMC7856196

[31] Santhekadur PK Kumar DP Sanyal AJ. Preclinical models of non-alcoholic fatty liver disease. J Hepatol (2018) 68(2):230–7. doi: 10.1016/j.jhep.2017.10.031 PMC5775040 29128391

[32] Yu R Zhang J Zhuo Y Hong X Ye J Tang S. Identification of diagnostic signatures and immune cell infiltration characteristics in rheumatoid arthritis by integrating bioinformatic analysis and machine-learning strategies. Front Immunol (2021) 12:724934. doi: 10.3389/fimmu.2021.724934 34691030 PMC8526926

[33] Luci C Bourinet M Leclere PS Anty R Gual P. Chronic inflammation in non-alcoholic steatohepatitis: molecular mechanisms and therapeutic strategies. Front Endocrinol (Lausanne) (2020) 11:597648. doi: 10.3389/fendo.2020.597648 33384662 PMC7771356

[34] Ficht X Iannacone M. Immune surveillance of the liver by T cells. Sci Immunol (2020) 5(51):eaba2351. doi: 10.1126/sciimmunol.aba2351 32887842

[35] Hirsova P Bamidele AO Wang H Povero D Revelo XS. Emerging roles of T cells in the pathogenesis of nonalcoholic steatohepatitis and hepatocellular carcinoma. Front Endocrinol (Lausanne) (2021) 12:760860. doi: 10.3389/fendo.2021.760860 34777255 PMC8581300

[36] Her Z Tan JHL Lim YS Tan SY Chan XY Tan WWS. Cd4(+) T cells mediate the development of liver fibrosis in high fat diet-induced nafld in humanized mice. Front Immunol (2020) 11:580968. doi: 10.3389/fimmu.2020.580968 33013934 PMC7516019

[37] Luo XY Takahara T Kawai K Fujino M Sugiyama T Tsuneyama K. Ifn-gamma deficiency attenuates hepatic inflammation and fibrosis in a steatohepatitis model induced by a methionine- and choline-deficient high-fat diet. Am J Physiol Gastrointest Liver Physiol (2013) 305(12):G891–9. doi: 10.1152/ajpgi.00193.2013 24136786

[38] Rau M Schilling AK Meertens J Hering I Weiss J Jurowich C. Progression from nonalcoholic fatty liver to nonalcoholic steatohepatitis is marked by a higher frequency of Th17 cells in the liver and an increased Th17/Resting regulatory T cell ratio in peripheral blood and in the liver. J Immunol (2016) 196(1):97–105. doi: 10.4049/jimmunol.1501175 26621860

[39] Lin Z Peng R Sun Y Zhang L Zhang Z. Identification of ribosomal protein family in triple-negative breast cancer by bioinformatics analysis. Biosci Rep (2021) 41(1):BSR20200869. doi: 10.1042/BSR20200869 33305312 PMC7789804

[40] Wang W Nag S Zhang X Wang MH Wang H Zhou J. Ribosomal proteins and human diseases: pathogenesis, molecular mechanisms, and therapeutic implications. Med Res Rev (2015) 35(2):225–85. doi: 10.1002/med.21327 PMC4710177 25164622

[41] Luan Y Tang N Yang J Liu S Cheng C Wang Y. Deficiency of ribosomal proteins reshapes the transcriptional and translational landscape in human cells. Nucleic Acids Res (2022) 50(12):6601–17. doi: 10.1093/nar/gkac053 PMC9262593 35137207

[42] Lindstrom MS. Emerging functions of ribosomal proteins in gene-specific transcription and translation. Biochem Biophys Res Commun (2009) 379(2):167–70. doi: 10.1016/j.bbrc.2008.12.083 19114035

[43] Li YH Wang J Liu Y Qiu L Li JZ Hu HG. Esculentoside a specifically binds to ribosomal protein S3a and impairs lps-induced signaling in macrophages. Int Immunopharmacol (2018) 54:254–60. doi: 10.1016/j.intimp.2017.11.018 29169044

[44] Kho CJ Zarbl H. Fte-1, a V-fos transformation effector gene, encodes the mammalian homologue of a yeast gene involved in protein import into mitochondria. Proc Natl Acad Sci U.S.A. (1992) 89(6):2200–4. doi: 10.1073/pnas.89.6.2200 PMC48624 1549582

[45] Slizhikova DK Vinogradova TV Sverdlov ED. [the Nola2 and Rps3a genes as highly informative markers for human squamous cell lung cancer]. Bioorg Khim (2005) 31(2):195–9. doi: 10.1007/s11171-005-0024-6 15889794

[46] Tarantul VZ Nikolaev AI Martynenko A Hannig H Hunsmann G Bodemer W. Differential gene expression in b-cell non-hodgkin's lymphoma of siv-infected monkey. AIDS Res Hum Retroviruses (2000) 16(2):173–9. doi: 10.1089/088922200309511 10659056

[47] Zhou C Weng J Liu C Zhou Q Chen W Hsu JL. High Rps3a expression correlates with low tumor immune cell infiltration and unfavorable prognosis in hepatocellular carcinoma patients. Am J Cancer Res (2020) 10(9):2768–84. PMC7539769 33042616

[48] Lim KH Kim KH Choi SI Park ES Park SH Ryu K. Rps3a over-expressed in hbv-associated hepatocellular carcinoma enhances the hbx-induced nf-kappab signaling *Via* its novel chaperoning function. PLoS One (2011) 6(8):e22258. doi: 10.1371/journal.pone.0022258 21857917 PMC3156704

[49] Tang Y He Y Li C Mu W Zou Y Liu C. Rps3a positively regulates the mitochondrial function of human periaortic adipose tissue and is associated with coronary artery diseases. Cell Discovery (2018) 4:52. doi: 10.1038/s41421-018-0041-2 30131868 PMC6102269

[50] Liu WJ Zhou L Liang ZY Zhou WX You L Zhang TP. Plasminogen activator inhibitor 1 as a poor prognostic indicator in resectable pancreatic ductal adenocarcinoma. Chin Med J (Engl) (2018) 131(24):2947–52. doi: 10.4103/0366-6999.247211 PMC6302640 30539907

[51] Chen R Dawson DW Pan S Ottenhof NA de Wilde RF Wolfgang CL. Proteins associated with pancreatic cancer survival in patients with resectable pancreatic ductal adenocarcinoma. Lab Invest (2015) 95(1):43–55. doi: 10.1038/labinvest.2014.128 25347153 PMC4281293

[52] Bi N Sun Y Lei S Zeng Z Zhang Y Sun C. Identification of 40s ribosomal protein S8 as a novel biomarker for Alcohol−Associated hepatocellular carcinoma using weighted gene Co−Expression network analysis. Oncol Rep (2020) 44(2):611–27. doi: 10.3892/or.2020.7634 PMC7336510 32627011

[53] Jiang N Hu L Liu C Gao X Zheng S. 60s ribosomal protein L35 regulates beta-casein translational elongation and secretion in bovine mammary epithelial cells. Arch Biochem Biophys (2015) 583:130–9. doi: 10.1016/j.abb.2015.08.006 26297660

[54] Liu PY Tee AE Milazzo G Hannan KM Maag J Mondal S. The long noncoding rna Lncnb1 promotes tumorigenesis by interacting with ribosomal protein Rpl35. Nat Commun (2019) 10(1):5026. doi: 10.1038/s41467-019-12971-3 31690716 PMC6831662

[55] Wu W Yu N Li F Gao P Lin S Zhu Y. Rpl35 promotes neuroblastoma progression *Via* the enhanced aerobic glycolysis. Am J Cancer Res (2021) 11(11):5701–14. PMC8640819 34873488

[56] Lin X Guo L Lin X Wang Y Zhang G. Expression and prognosis analysis of mitochondrial ribosomal protein family in breast cancer. Sci Rep (2022) 12(1):10658. doi: 10.1038/s41598-022-14724-7 35739158 PMC9226049

[57] Ueta M Nishigaki H Komai S Mizushima K Tamagawa-Mineoka R Naito Y. Positive regulation of innate immune response by mirna-Let-7a-5p. Front Genet (2022) 13:1025539. doi: 10.3389/fgene.2022.1025539 36685889 PMC9858567

[58] Peng L Chen Y Ou Q Wang X Tang N. Lncrna miat correlates with immune infiltrates and drug reactions in hepatocellular carcinoma. Int Immunopharmacol (2020) 89(Pt A):107071. doi: 10.1016/j.intimp.2020.107071 33221703

[59] Zhang X Pan B Qiu J Ke X Shen S Wang X. Lncrna miat targets mir-411-5p/Stat3/Pd-L1 axis mediating hepatocellular carcinoma immune response. Int J Exp Pathol (2022) 103(3):102–11. doi: 10.1111/iep.12440 PMC9107606 35429078

[60] He R Chen Y Chen X Yuan B. Mechanism of mir-181a-5p in regulatory T/T-helper 17 immune imbalance and asthma development in mice with allergic rhinitis. Int Arch Allergy Immunol (2022) 183(4):375–88. doi: 10.1159/000519703 34942624

[61] Lu J Li S Li X Zhao W Duan X Gu X. Declined mir-181a-5p expression is associated with impaired natural killer cell development and function with aging. Aging Cell (2021) 20(5):e13353. doi: 10.1111/acel.13353 33780118 PMC8135006

[62] Zhan ZY Wu M Shang Y Jiang M Liu J Qiao CY. Taxifolin ameliorate high-Fat-Diet feeding plus acute ethanol binge-induced steatohepatitis through inhibiting inflammatory caspase-1-Dependent pyroptosis. Food Funct (2021) 12(1):362–72. doi: 10.1039/d0fo02653k 33325949

[63] Inoue T Fu B Nishio M Tanaka M Kato H Tanaka M. Novel therapeutic potentials of taxifolin for obesity-induced hepatic steatosis, fibrogenesis, and tumorigenesis. Nutrients (2023) 15(2):350. doi: 10.3390/nu15020350 36678220 PMC9865844

[64] Zhou ST Cui W Kong L Yang X. Efficacy of sitagliptin on nonalcoholic fatty liver disease in high-Fat-Diet-Fed diabetic mice. Curr Med Sci (2022) 42(3):513–9. doi: 10.1007/s11596-022-2573-9 35451807

[65] Shen T Xu B Lei T Chen L Zhang C Ni Z. Sitagliptin reduces insulin resistance and improves rat liver steatosis *Via* the Sirt1/Ampkalpha pathway. Exp Ther Med (2018) 16(4):3121–8. doi: 10.3892/etm.2018.6554 PMC6125831 30214535

[66] Yan J Yao B Kuang H Yang X Huang Q Hong T. Liraglutide, sitagliptin, and insulin glargine added to metformin: the effect on body weight and intrahepatic lipid in patients with type 2 diabetes mellitus and nonalcoholic fatty liver disease. Hepatology (2019) 69(6):2414–26. doi: 10.1002/hep.30320 PMC6594101 30341767

