# Supplementary material for: Identification of ribosomal protein family as immune-cell-related biomarkers of NAFLD by bioinformatics and experimental analyses
Source: Front Endocrinol (Lausanne). 2023 May 19;14:1161269. doi: 10.3389/fendo.2023.1161269 (PMC10235545; doi:10.3389/fendo.2023.1161269)
Supplement: Supplementary file 1 [file DataSheet_1.docx]

Supplementary Material

**Identification of ribosomal protein family as immune-cell-related biomarkers of NAFLD by bioinformatics and experimental analyses**

Gerui Li^1,†^, Hang Li^1,†^, Ze Chen^2,3,^*

^1^Department of Geriatrics, Zhongnan Hospital of Wuhan University, Wuhan 430071, China;

^2^Department of Cardiology, Zhongnan Hospital of Wuhan University, Wuhan 430071, Hubei, China;

^3^Institute of Myocardial Injury and Repair, Wuhan University, Wuhan 430071, China.

^†^Gerui Li and Hang Li contributed equally to this work and share first authorship

*** Correspondence:** Ze Chen: chenze19@whu.edu.cn

# Supplementary Figures and Tables

## Supplementary Figures

**
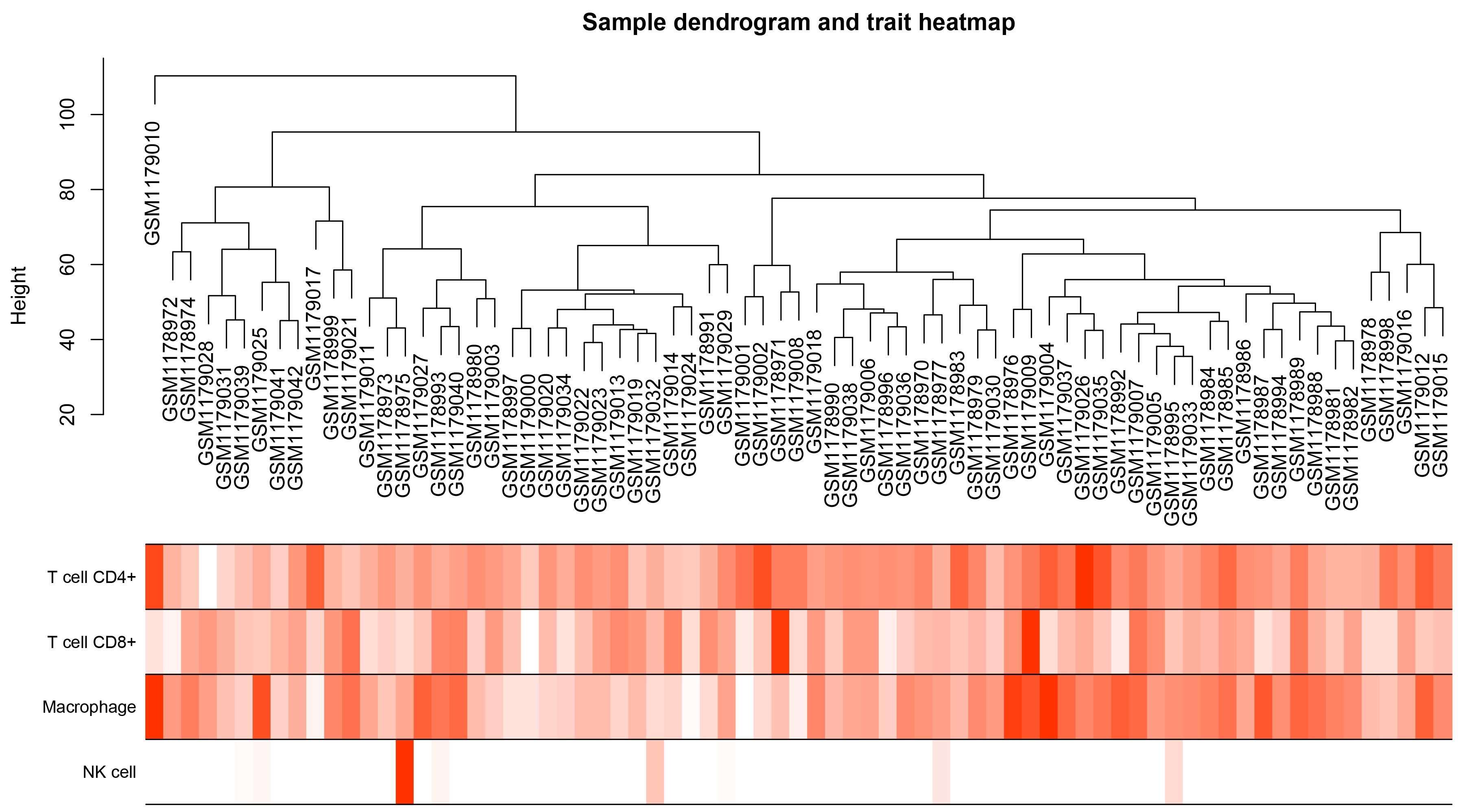
Supplementary Figure 1.** Sample dendrogram and trait heatmap.

**
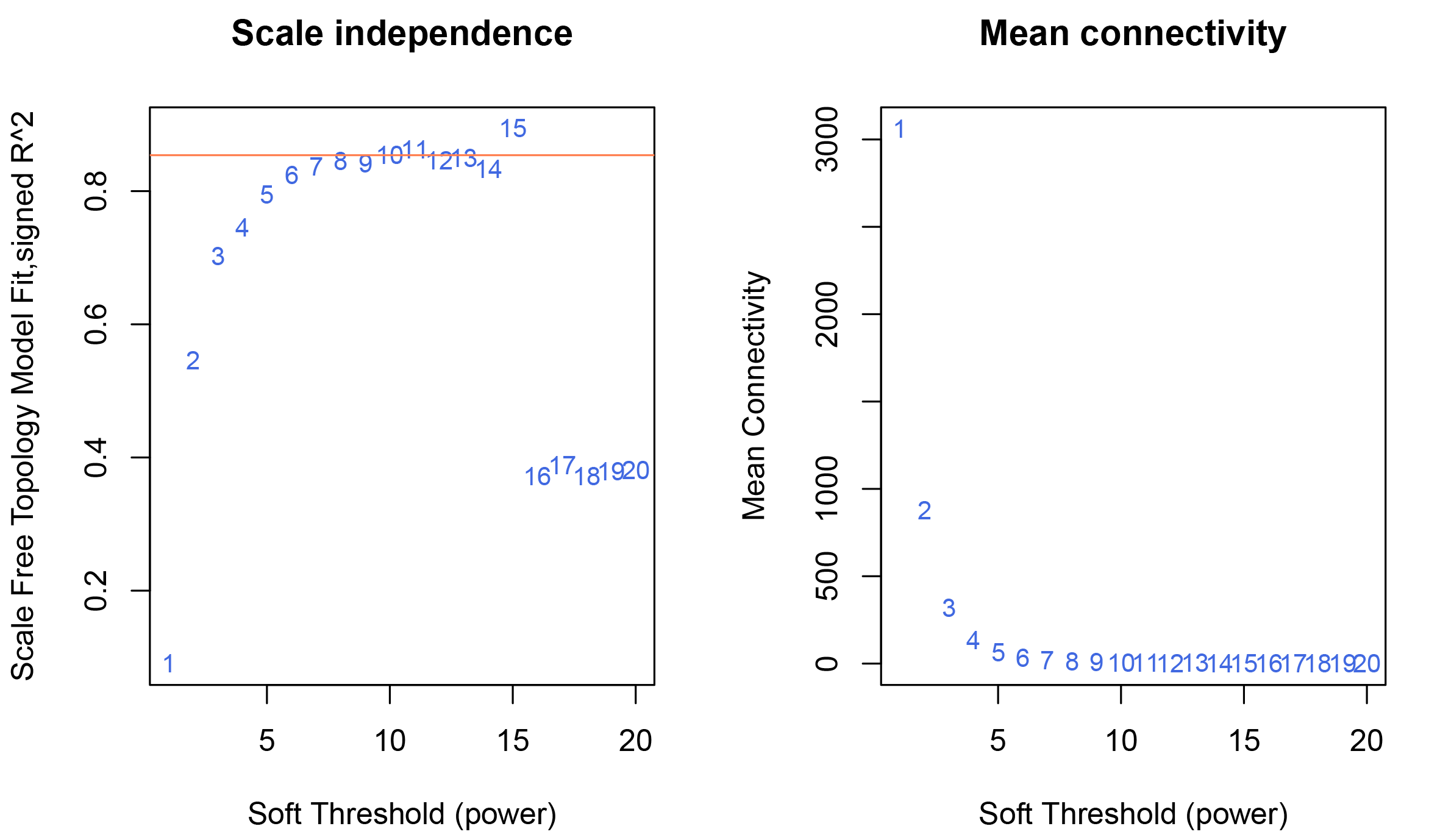
**

**Supplementary Figure 2.** Analysis of network topology for various soft-thresholding powers.

**
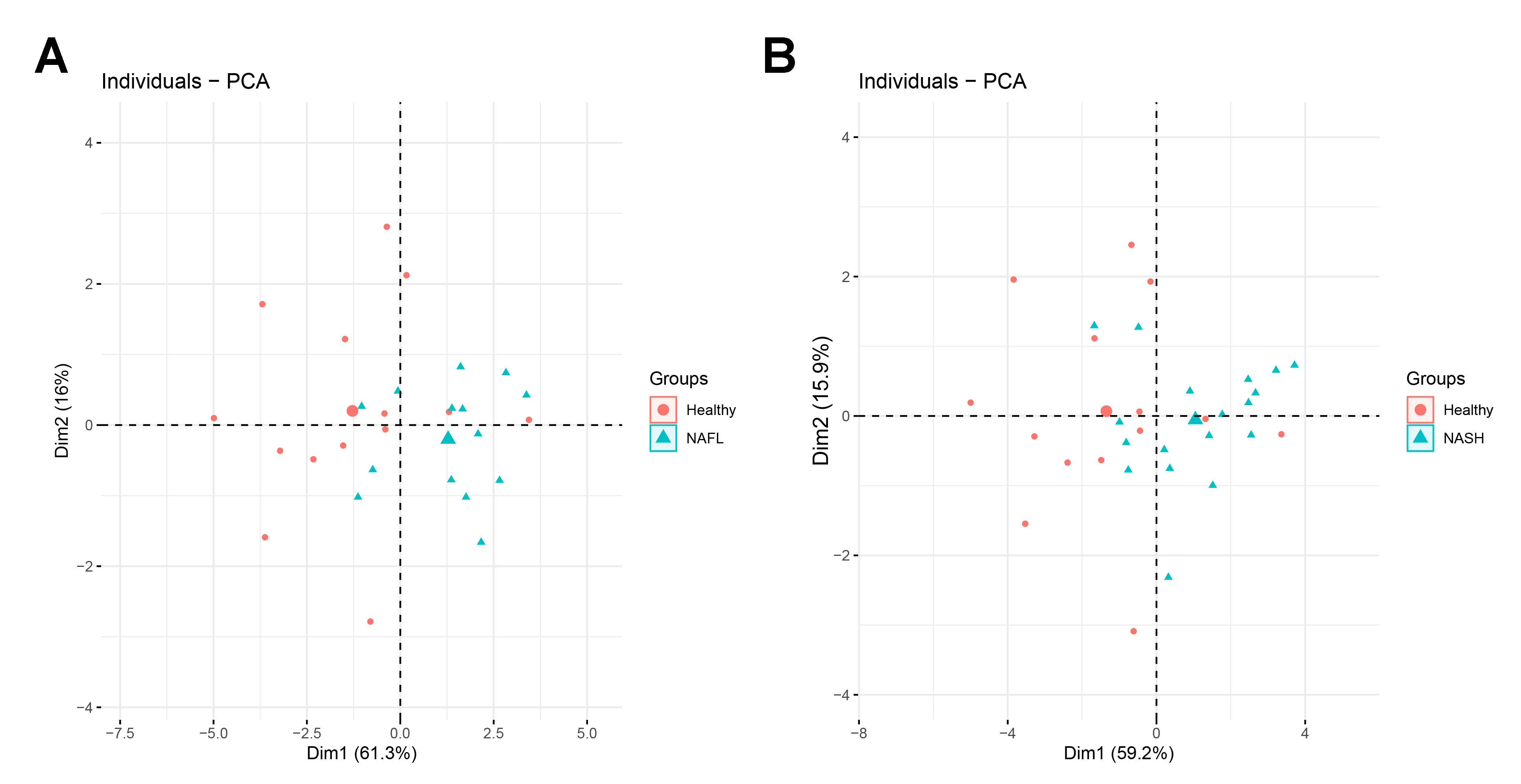
**

**Supplementary Figure 3.** Principal component analysis (PCA) based on the expression of 9 hub genes. PCA distinguishing the healthy and NAFL samples (A) as well as the healthy and NASH samples (B) in dataset GSE48452.


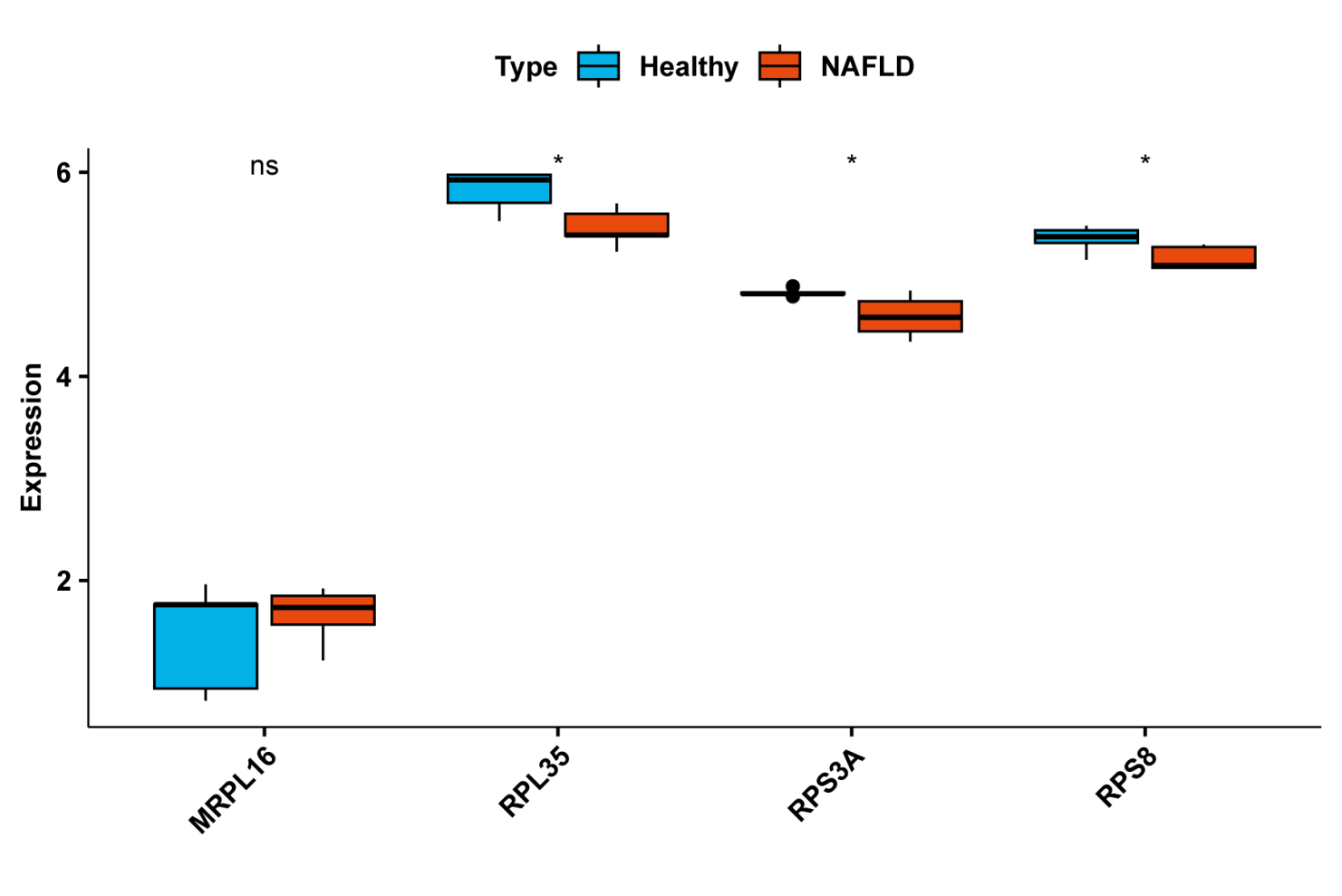


**Supplementary Figure 4.** External validation of the expression of immune-cell-related biomarkers in dataset GSE107231.

## Supplementary Tables

**Supplementary Table 1. The topological structure of hub genes.**

| **Node name** | **MCC** | **DMNC** | **MNC** | **Degree** | **EPC** |
| --- | --- | --- | --- | --- | --- |
| FCGR2B | 41 | 0.34989 | 8 | 9 | 9.89 |
| IL2RB | 38 | 0.40246 | 7 | 7 | 9.598 |
| RPL17 | 60 | 0.57059 | 6 | 6 | 7.584 |
| RPL35 | 60 | 0.57059 | 6 | 6 | 7.588 |
| INPP5D | 11 | 0.32413 | 5 | 6 | 9.015 |
| RPS8 | 50 | 0.47549 | 6 | 6 | 7.506 |
| RPS3A | 50 | 0.47549 | 6 | 6 | 7.575 |
| MRPL16 | 31 | 0.51861 | 5 | 6 | 7.564 |
| RPL10 | 30 | 0.51861 | 5 | 5 | 7.351 |
| CD40LG | 26 | 0.45378 | 5 | 5 | 8.863 |
| CD247 | 30 | 0.51861 | 5 | 5 | 9.234 |
| CD74 | 5 | 0.30779 | 2 | 5 | 7.582 |
| CCR7 | 26 | 0.45378 | 5 | 5 | 9.18 |

MCC, maximal clique centrality; DMNC, density of maximum neighborhood component; MNC, maximum neighborhood component; EPC, edge percolated component.

**Supplementary Table 2. Performance of hub genes in ROC curve analysis in datasets GSE48452 and GSE126848.**

| **Gene name** | **GSE48452** | | | **GSE126848** | | |
| --- | --- | --- | --- | --- | --- | --- |
|  | **AUC** | **Sensitivity** | **Specificity** | **AUC** | **Sensitivity** | **Specificity** |
| CD247 | 0.71 | 0.57 | 0.81 | 0.90 | 0.86 | 0.81 |
| CD74 | 0.76 | 0.43 | 1.00 | 0.95 | 1.00 | 0.84 |
| FCGR2B | 0.73 | 0.71 | 0.81 | 0.90 | 1.00 | 0.71 |
| IL2RB | 0.74 | 0.86 | 0.66 | 0.97 | 1.00 | 0.87 |
| INPP5D | 0.74 | 0.50 | 0.88 | 1.00 | 1.00 | 0.97 |
| MRPL16 | 0.75 | 0.64 | 0.94 | 0.85 | 0.79 | 0.94 |
| RPL35 | 0.75 | 0.79 | 0.78 | 0.91 | 0.86 | 0.97 |
| RPS3A | 0.84 | 0.79 | 0.78 | 0.85 | 0.79 | 0.84 |
| RPS8 | 0.80 | 0.86 | 0.75 | 0.86 | 0.79 | 0.94 |

AUC, area under the curve.

**Supplementary Table 3. Potential drugs for the treatment of NAFLD.**

| **Gene** | **Score** | **Name** | **Description** |
| --- | --- | --- | --- |
| RPS3A | 98.13 | otenzepad | acetylcholine receptor antagonist |
| RPS3A | 98.12 | famotidine | histamine receptor antagonist |
| RPS3A | 97.38 | JNJ-7706621 | CDK inhibitor, Aurora kinase inhibitor |
| RPS3A | 96.92 | nisoldipine | calcium channel blocker, L-type calcium channel blocker |
| RPS3A | 96.62 | ampiroxicam | cyclooxygenase inhibitor |
| RPS3A | 96.51 | amoxicillin | cell wall synthesis inhibitor, PBPA inhibitor |
| RPS3A | 96.08 | daunorubicin | RNA synthesis inhibitor, topoisomerase inhibitor, DNA synthesis inhibitor, radical formation stimulant |
| RPS3A | 95.79 | taxifolin | apolipoprotein secretion inhibitor, beta amyloid aggregation inhibitor, cholesterol biosynthesis inhibitor, HMGCR inhibitor, NFkB pathway modulator, opioid receptor antagonist, reverse transcriptase inhibitor |
| RPS3A | 94.73 | oxcarbazepine | voltage-gated sodium channel blocker, Sodium Channel Blockers |
| RPS3A | 94.72 | staurosporine | PKC inhibitor, AKT inhibitor, BMX inhibitor, CDK inhibitor, CHK inhibitor, G protein coupled receptor agonist, glycogen synthase kinase inhibitor, leucine rich repeat kinase inhibitor, ribosomal protein inhibitor, sodium/hydrogen exchanger inhibitor |
| RPS3A | -32.98 | cholic-acid | ferrochelatase inhibitor, unidentified pharmacological activity |
| RPS3A | -35.3 | mupirocin | isoleucyl-tRNA synthetase inhibitor |
| RPS3A | -38.68 | ebelactone-b | pancreatic lipase inhibitor |
| RPS3A | -49.65 | SJ-172550 | MDM inhibitor |
| RPS3A | -49.69 | androstenol | GABA receptor modulator |
| RPS3A | -52.64 | mefenamic-acid | cyclooxygenase inhibitor |
| RPS3A | -55.5 | carbetocin | analogue of oxytocin |
| RPS3A | -65.74 | deforolimus | mTOR inhibitor, angiogenesis inhibitor, cell cycle inhibitor, immunosuppressant, protein kinase inhibitor, serine/threonine kinase inhibitor, VEGFR antagonist |
| RPS3A | -68.49 | BAS-09104376 | HIV integrase inhibitor |
| RPS3A | -73.94 | sitagliptin | dipeptidyl peptidase inhibitor, HMGCR inhibitor, insulin secretagogue, tumor necrosis factor expression inhibitor |

**Supplementary Table 4. Biochemical and histological characteristics of NAFLD mouse model.**

| **Parameters** | **NC** | | **HFD** | | ***P* value** |
| --- | --- | --- | --- | --- | --- |
|  | **Mean** | **S.E.M** | **Mean** | **S.E.M** |  |
| Serum ALT (IU/L) | 42.2 | 4.4 | 87.8 | 9.8 | < 0.01 |
| Hepatic TG (mg/g) | 29.6 | 5.0 | 113.5 | 13.1 | < 0.01 |
| NAFLD activity score | 1.2 | 0.3 | 4.5 | 0.3 | < 0.01 |

NC, normal chow; HFD, high-fat diet; S.E.M, standard error of the mean; ALT, alanine aminotransferase; TG, triglyceride.
